# Evaluation of a Mobile Phone–Based Intervention to Increase Parents’ Knowledge About the Measles-Mumps-Rubella Vaccination and Their Psychological Empowerment: Mixed-Method Approach

**DOI:** 10.2196/mhealth.8263

**Published:** 2018-03-07

**Authors:** Marta Fadda, Elisa Galimberti, Maddalena Fiordelli, Peter Johannes Schulz

**Affiliations:** ^1^ Institute of Communication and Health University of Lugano Lugano Switzerland; ^2^ Health Ethics and Policy Lab Department of Health Sciences and Technology ETH Zurich Zurich Switzerland

**Keywords:** qualitative research, measles-mumps-rubella vaccine, surveys and questionnaires, mobile applications, knowledge, patient participation

## Abstract

**Background:**

There is mixed evidence on the effectiveness of vaccination-related interventions. A major limitation of most intervention studies is that they do not apply randomized controlled trials (RCTs), the method that, over the last 2 decades, has increasingly been considered as the only method to provide proof of the effectiveness of an intervention and, consequently, as the most important instrument in deciding whether to adopt an intervention or not. This study, however, holds that methods other than RCTs also can produce meaningful results.

**Objective:**

The aim of this study was to evaluate 2 mobile phone–based interventions aimed at increasing parents’ knowledge of the measles-mumps-rubella (MMR) vaccination (through elements of gamification) and their psychological empowerment (through the use of narratives), respectively. The 2 interventions were part of an RCT.

**Methods:**

We conducted 2 studies with the RCT participants: a Web-based survey aimed at assessing their rating of the tool regarding a number of qualities such as usability and usefulness (N=140), and qualitative telephonic interviews to explore participants’ experiences with the app (N=60).

**Results:**

The results of the survey showed that participants receiving the knowledge intervention (alone or together with the empowerment intervention) liked the app significantly better compared with the group that only received the empowerment intervention (*F*_2,137_=15.335; *P*<.001). Parents who were exposed to the empowerment intervention complained that they did not receive useful information but were only invited to make an informed, autonomous MMR vaccination decision.

**Conclusions:**

The results suggest that efforts to empower patients should always be accompanied by the provision of factual information. Using a narrative format that promotes parents’ identification can be an appropriate strategy, but it should be employed together with the presentation of more points of views and notions regarding, for instance, the risks and benefits of the vaccination at the same time.

**Trial Registration:**

International Standard Randomized Controlled Trial Number 30768813; http://www.isrctn.com/ ISRCTN30768813 (Archived by WebCite at http://www.webcitation.org/6xOQSJ3w8)

## Introduction

### Background

Childhood vaccination coverage is generally high in most developed countries, but clusters of individuals who remain unvaccinated (eg, because they share inaccurate beliefs about one or more immunizations) indicate that the phenomenon of vaccine hesitancy remains a significant problem [1]. It includes not only refusing some or all recommended vaccinations but also accepting them despite doubt and uncertainty. To decrease vaccine hesitancy, a number of interventions employing different designs and based on various frameworks have been proposed [2-8]. Sadaf and colleagues summarized such interventions into 3 groups: (1) passage of state laws (such as school immunization requirements), (2) state- and school-level implementation of laws (procedural complexities of obtaining nonmedical exemptions and school policies for immunization requirements), and (3) parent-centered immunization interventions, generally with information or education purposes [8]. Williams divided the latter type of interventions into different strategies to improve (1) parental attitudes about childhood vaccines, (2) vaccination intent, or (3) vaccination uptake among vaccine-hesitant parents [9]. More recently, Willis and colleagues have proposed a classification that includes 7 main categories that can be used in communication interventions targeting parents or soon-to-be-parents, community members, and health care providers: inform or educate, remind or recall, teach skills, provide support, facilitate decision making, enable communication, and enhance community ownership [10].

A recent review concluded that there is mixed evidence on the effectiveness of vaccination-related interventions involving face-to-face communication interventions, health care provider training, community-based actions, or communication using mass media [2]. A major limitation of most interventions is that they lack a rigorous evaluative assessment [2]. In fact, over the last 2 decades, randomized controlled trials (RCTs) have been increasingly considered as the gold standards in evidence-based practice, the only way to prove the effectiveness of an intervention and, consequently, as the most important instrument in deciding whether to adopt an intervention or not [11]. According to their supporters, RCTs have great ability “to minimize selection and information bias, control confounding, and for ruling out chance” [11]. At the same time, however, RCTs might not be enough to achieve results that are useful in practice [11]. In particular, many of the most important issues faced by RCT participants—their feelings, hopes, and beliefs, for example—cannot be meaningfully reduced to numbers or adequately understood without reference to the immediate context in which they live [12]. Consequently, RCTs are called for that are supplemented by research components that are either qualitative or the combination of qualitative and quantitative methods [11]. This strategy can provide evidence about how the intervention works (or why it did not), for whom, and under what circumstances [12].

Between December 1, 2016 and 10, 2016 our research team delivered 2 immunization interventions through a mobile phone app as an RCT [13]. The app, called MorbiQuiz, is in Italian language and can be downloaded free of charge in the Italian and Swiss Google Play and App Store. In the first intervention, aimed at increasing participants’ knowledge about the measles-mumps-rubella (MMR) vaccination using gamification, participants received 35 questions distributed on a time span of 10 days (3-4 questions per day). Once answered, each question unblocked an explanation of the answer through textual content. Each correct answer would earn participants a number of points (stars) according to the weight of each question, whereas no points were given for wrong answers or if no answer was given by midnight of the day. To provide a gamified experience, participants could see their score and compare it with that of the other participants through a leaderboard. Furthermore, participants were awarded a shopping voucher, which increased their performance in the quiz. The design of the app is extensively described in the paper reporting the results of the RCT [13].

In the second intervention, aimed at enhancing psychological empowerment (defined as a set of 4 subdimensions: self-determination, self-efficacy, impact, and meaningfulness), users received 2 videos and 8 messages. In the 2 videos, an actress acting as a mother reports that she was able to make an empowered decision about the MMR vaccination by collecting reliable information from multiple sources, and by thinking about the importance and the impact of the decision. In the end, she addresses her audience, encouraging them to make an informed, empowered decision. The viewer was addressed in the second person to increase participant involvement. The messages were designed to reinforce the messages delivered in the video. Participants received either the first, the second, or both interventions. A control group did not receive any intervention.

The effect of the 2 interventions (combined and alone) was tested on a number of outcomes such as vaccination knowledge, psychological empowerment, intention to vaccinate, confidence in the vaccination decision, vaccination opinion, intention to recommend the vaccination, and control preference in the vaccination decision making. All experimental groups reported a significant increase in their vaccination knowledge compared with the control (*F*_3,179_=48.58, *P*<.001), whereas only those participants who received both interventions reported a significant increase in their psychological empowerment (*t*_179_=-2.79, *P*=.006). Only those participants receiving the knowledge intervention had a significantly higher intention to vaccinate (*t*_179_=2.111; *P*=.03) and more confidence in the decision (*t*_179_=2.76; *P*=.006) compared with the control group.

As the experiment was only partially successful, we decided to assess the perceptions of the participants on a number of characteristics of the app and explore their experience with this tool. The effectiveness of the majority of vaccination interventions using new media, such as immunization apps, is simply evaluated looking at statistics regarding their download and usage [14-16]. These evaluative methods, however, provide no insights into participants’ perceptions regarding, for instance, the usability of the target tool. Furthermore, evaluations might be useful not only to collect participants’ perceptions but also to assess quantitative findings related to the intervention efficacy or explain why certain features did not have a significant effect on a given outcome.

### Objectives

The broader scope of this study is to evaluate 2 interventions administered through a mobile phone app [13]. The 2 interventions aimed at increasing parents’ knowledge of the MMR vaccination and their psychological empowerment, respectively, and were part of an RCT conducted in December 2016. Our 2 main research questions are as follows:

1. How did participants perceive the app’s usability and usefulness?
2. What was their experience with the tool and its functionalities?

To answer these questions, we conducted 2 studies with the RCT participants and employed a mixed-method approach. study 1 describes a Web-based survey aimed at quantifying participants’ rating of the tool regarding different qualities, including usability and usefulness, whereas study 2 takes the shape of a qualitative exploration of participants’ experiences with the app and of their feelings related to its use. The results of these studies will be interpreted in light of the quantitative results of the RCT, and practical implications for the design of future mobile phone–based immunization interventions will be discussed.

## Methods

### Study 1

Study 1 takes the shape of a Web-based survey that was included within the posttest questionnaire we sent via email or WhatsApp to the participants immediately after the end of the experiment. To be included in study 1, participants had to have at least 1 child younger than 15 months, to be a resident in the Lombardy region of Italy, and to own a mobile phone with Internet connection. We added following 2 exclusion criteria: being in the control group (participants who did not receive the app) and not having logged in on the app during the experiment. Recruitment of the participants for the experiment was conducted through registered pediatricians and a marketing agency between April and November 2016. Data were collected between December 11, 2016 and January 15, 2017. Informed consent was obtained before filling out the Web-based questionnaire, where a short paragraph informed participants about the length of time of the survey, which data would be stored, where and for how long, who the investigators were, the general purpose of the study, and that all answers would be analyzed to respect participants’ privacy and confidentiality. Participants’ could not change the answers provided.

### Measures

#### Mobile App Rating Scale

The Mobile App Rating Scale (MARS) is a 23-item scale developed to assess the quality of mobile health apps [17]. In previous studies, the scale showed high reliability [17,18]. The MARS is composed of 2 subscales, one assessing 4 objective qualities (engagement, functionality, aesthetics, and information quality) and the other assessing subjective qualities [17]. In addition, it provides 6 app-specific items measuring perceived outcomes to be adjusted to each health context [17]. The original scale was adapted to the context of our mobile phone app and included 8 items assessing all aforementioned 4 objective qualities and 2 items assessing subjective qualities. The objective qualities included entertainment, interest, interactivity, ease of use, visual appeal, goals, quality of information, and credibility. They were all measured with 1 item each, and response was recorded on a 5-point Likert scale measuring agreement and anchoring at “Absolutely agree” and “Absolutely disagree.” To measure the app’s subjective qualities we included a star-rating question (with the possible scores ranging from 1 to 5 stars) and one question asking how likely the participant would recommend the app in the future (with answers ranging from “Very unlikely” to “Very likely” on a 5-point scale).

In addition, we included 3 items assessing participants’ perceived impact of the app on their knowledge (MorbiQuiz has helped me deepen my knowledge of vaccination), on their help seeking (MorbiQuiz has increased my desire to collect information about vaccination), and the perceived likelihood of an actual change in the target health behavior (After using MorbiQuiz, do you think that this app could change parents’ vaccination decision?). Responses were recorded on a 5-point scale measuring agreement and anchoring at “Absolutely agree” and “Absolutely disagree” for the first 2 items, whereas they were measured on a 7-point scale ranging from “Yes, discouraging vaccination” to “Yes, favoring vaccination” for the third item. A midway option “I don’t think it can make a difference” was also provided.

The posttest questionnaire also assessed the experiment’s primary and secondary variables measured in the baseline survey (intention to vaccinate, confidence in the decision, etc), participants’ social norms regarding the MMR vaccination decision, any problems that prevented a regular access to the app during the experiment, and participants’ Web-based information-seeking behaviors. A pretest took place before sending the questionnaire to the participants to ensure content validity.

#### Sociodemographic Information

We assessed a number of sociodemographic characteristics, including gender, age, education, nationality, number of children, and ZIP code.

#### Analyses

Participants’ responses were captured automatically, and data analysis was performed using the Statistical Package for Social Science (IBM Corp, version 21.0). Analysis of variances (ANOVAs) were performed for each variable to determine whether there were differences among the experimental conditions. Where appropriate, planned contrasts were conducted to analyze significant differences across the experimental conditions.

### Study 2

Study 2 is a qualitative study conducted with a subsample of the participants who took part in study 1. Participants were recruited through the posttest questionnaire that followed the assessment of the experiment. To recruit participants, a final question was added to the questionnaire, asking whether we could contact the participant for a short telephonic interview to share the experience with the app. A lottery was employed as an incentive to participation, with one shopping voucher worth 200 euros to be drawn. If participants accepted to be contacted, they were asked to provide a telephone number. We sent a message to all telephone numbers provided, asking to suggest a suitable date and time when to conduct the interview. We developed a list of semistructured interview questions aimed at exploring the perceptions and experiences of parents with regard to their use of the app (see Multimedia Appendix 1). All questions were open-ended to facilitate our understanding of parents’ experiences and feelings, as well as their suggestions and remarks. The interview grid was flexible in the sense that the question order could be changed according to the flow of the conversation. Consent to participate and to have the interview recorded was obtained before starting the interview. We recorded all interviews using a call recorder app and transcribed them verbatim.

Inductive thematic analysis of the transcripts was conducted independently by 2 coders [19]. Initially, the transcripts were read several times and openly coded manually, underlying meaningful parts. At a later stage, all codes were grouped under labels and organized hierarchically using a tree diagram. All labels were finally grouped under broader themes. During the whole process, telephonic and face-to-face meetings between the 2 coders were regularly conducted to compare, discuss, and refine the codes, labels, preliminary themes, and relative quotations. We conducted the interviews between December 19, 2016 and January 13, 2017. Both the transcription and the analysis of the interviews were conducted in the original language (Italian).

## Results

### Study 1

#### Participants’ Characteristics

In total, 140 participants of the RCT answered questions related to the app’s qualities, representing all the participants in the 3 experimental groups of the RCT [13]. The majority of the participants had only 1 child (n=110), were mothers (n=138), and Italian nationals (n=136). Participants’ mean age was 33.96 (standard deviation [SD]=5.52, range=21-47). About one-third had completed secondary school (n=43), whereas most had a university degree (n=84). See Table 1 for participants’ characteristics and Table 2 for their scores related to the app’s qualities.

**Table 1:** Participants’ characteristics (study 1, N=140).

Characteristic		Experimental group		
		1: Quiz only (n=48)	2: Videos and messages only (n=45)	3 Quiz + Videos and messages (n=47)
**Gender, n (%)**				
	Women	43 (90)	43 (96)	46 (98)
	Men	5 (10)	2 (4)	1 (2)
Age, mean (SD)		33.44 (4.27)	34.49 (4.46)	33.98 (4.86)
**Nationality, n (%)**				
	Italian	45 (94)	45 (100)	46 (98)
	Brazilian	N/A^a^	N/A	1 (2)
	Mexican	1 (2)	N/A	N/A
	Moroccan	1 (2)	N/A	N/A
**Education, n (%)**				
	Middle School	3 (6)	N/A	1 (2)
	University	23 (48)	30 (67)	31 (66)
	Secondary School	17 (35)	13 (29)	13 (28)
	Apprentice	4 (8)	2 (4)	2 (4)
**No. of children, n (%)**				
	1	40 (84)	35 (78)	35 (74)
	2 or more	8 (16)	10 (22)	12 (26)

^a^N/A: Not applicable.

**Table 2:** Survey results per experimental group.

Quality		Survey item	Experimental group			*F* (degrees of freedom); *P* value	Posthoc test^a^
			1: Quiz only (n=48), mean (SD)	2: Videos and messages only (n=45), mean (SD)	3: Quiz + Videos and messages (n=47), mean (SD)				
**Engagement**							
	Entertainment	Using MorbiQuiz was fun	4.63 (0.57)	3.87(0.94)	4.62 (0.79)	14.248 (2,137); *P*<.001	13 2
	Interest	The contents of MorbiQuiz are presented in an interesting way	4.54 (0.74)	3.78 (1.02)	4.45 (0.90)	9.97 (2,137); *P*<.001	13 2
	Interactivity	I felt as Sofia was talking to me	N/A^b^	3.80 (1.25)	3.72 (1.19)	0.09 (1,90); *P*=.76	N/A
**Functionality**							
	Ease of use	MorbiQuiz is easy to use	4.81 (0.44)	4.20 (1.01)	4.70 (0.75)	8.35 (2,137); *P*<.001	13 2
**Aesthetics**							
	Visual appeal	I like the graphics of MorbiQuiz	4.65 (1.635)	4.07 (1.03)	4.55 (0.829)	6.252 (2,137); *P*=.003	13 2
**Information**							
	Goals	It is easy to understand what MorbiQuiz is for	4.63 (0.61)	4.20 (0.84)	4.70 (0.55)	7.36 (2,137); *P*=.001	31 2
	Quality of information	MorbiQuiz’s contents are easy to understand	4.56 (0.58)	4.40 (0.86)	4.36 (0.89)	0.86 (2,137); *P*=.42	N/A
	Credibility	The contents of the quiz are reliable	4.5 (0.62)	N/A	4.49 (0.8)	0.005 (1,90); *P*=.94	N/A
		The contents of the videos are reliable	N/A	4.11 (0.86)	4.23 (0.96)	0.42 (1,90); *P*=.52	N/A
**Subjective**							
	Star rating	How would you rate MorbiQuiz?	4.5 (0.55)	3.76 (0.74)	4.23 (0.67)	15.335 (2,137); *P*<.001	13 2
	Future recommendation	How likely are you to recommend MorbiQuiz to other parents?	4.27 (0.87)	3.91 (0.7)	4.38 (0.79)	4.419 (2,137); *P*=.01	3 2
**App specific**							
	Awareness/knowledge	MorbiQuiz has helped me deepen my knowledge of vaccination	4.58 (0.58)	3.89 (0.88)	4.70 (0.72)	16.36 (2,137); *P*<.001	31 2
	Help seeking	MorbiQuiz has increased my desire to collect information about vaccination	4.42 (0.68)	4.09 (0.90)	4.34 (0.91)	1.93 (2,137); *P*=.15	N/A
	Behavior change	After using MorbiQuiz, do you think that this app could change parents’ vaccination decision?	—	—	—	—	—

^a^Close groups significantly differ from other.

^b^N/A: Not applicable.

#### Objective Qualities

Participants’ scores related to the app’s objective qualities were, overall, high. We found, however, significant differences among the 3 experimental groups for a number of qualities assessed.

##### Engagement

We found significant differences among the 3 groups regarding entertainment (*F*_2,137_=14.248; *P*<.001) and interest (*F*_2,137_=9.97; *P*<.001). In particular, participants who received the knowledge intervention, were more likely to report that using MorbiQuiz was fun (mean 4.63 [SD 0 .57]) and that the contents of MorbiQuiz were presented in an interesting way (mean 4.53 [SD 0 .74]) than respondents who had received the empowerment intervention (mean 3.87 [SD 0.94] and mean 3.78 [SD 1.02]). To understand what gamification adds to the perception of the intervention employing the videos, we also compared the groups receiving the empowerment intervention only with those receiving the combined version. Those in the combined intervention group also scored significantly more on entertainment (mean 4.62 [SD 0.79]) and interest (mean 4.45 [SD 0.90]). Concerning interactivity, which indicates the perception that Sofia (the mother acting in the 2 videos) was directly addressing the participant, we found no statistical difference between the empowerment intervention only and the combined interventions groups (*F*_1,90_=0.09; *P=*.76).

##### Functionality

The 3 experimental groups also significantly differed in their opinion on the extent to which MorbiQuiz is easy to use (*F*_2,137_=8.35; *P<*.001). Participants in the group receiving the knowledge intervention reported significantly higher ease of use of the app (mean 4.81 [SD 0.44]) compared with those who received the empowerment intervention (mean 4.20 [SD 1.01]). When we compared the groups receiving the empowerment intervention only with those who received both intervention, we found that the former reported significantly higher ease of use of the app compared with the latter (mean 4.70 [SD 0.75]).

##### Aesthetics

The 3 groups also showed significant differences in their perceived visual appeal of MorbiQuiz (*F*_2,137_=6.252; *P=*.003). Participants in the group receiving the knowledge intervention only reported significantly higher appreciation of the graphics of MorbiQuiz (mean 4.65 [SD 1.635]) compared with those who received the empowerment intervention (mean 4.07 [SD 1.03]). Participants in the group receiving the knowledge and empowerment interventions combined also reported significantly higher appreciation of the graphics of MorbiQuiz compared with those who received the empowerment intervention only (mean 4.55 [SD 0.829]).

##### Information

Regarding information, we found a statistical difference among experimental groups for goals (*F*_2,137_=7.36; *P=*.001) but not for the perceived quality (*F*_2,137_=0.86; *P=*.42) and credibility of the information (contents of the quiz: *F*_1,90_=0.005; *P=*.94; contents of the videos and messages: *F*_1,90_=.42; *P=*.52). In particular, participants in the groups receiving the knowledge intervention reported significantly higher ease in understanding the scope of MorbiQuiz (mean 4.63 [SD 0.61]) compared with those who received the empowerment intervention only (mean 4.20 [SD 0.84]). Those in the knowledge and empowerment interventions combined also reported significantly higher ease in understanding the scope of MorbiQuiz (mean 4.70 [SD 0 .55]) compared with those who received the empowerment intervention only.

### Subjective Qualities

Similar to the objective qualities, the app received high scores for the subjective qualities, with significant differences between experimental groups. In terms of rating (*F*_2,137_=15.335; *P<*.001), the groups receiving the knowledge intervention only gave MorbiQuiz a significant higher number of stars (mean 4.5 [SD 0.55]) compared with those who received the empowerment intervention only (mean 3.76 [SD 0.74]). Likewise, those in the knowledge and empowerment interventions combined gave MorbiQuiz a significant higher number of stars (mean 4.23 [SD 0.67]) compared with those who received the empowerment intervention only.

In general, disregarding the experimental group, parents would recommend the app (mean 4.19 [SD 0.813]). There are, however, statistically significant differences according to the experimental group (*F*_2,137_=4.419; *P=*.01). Those in the combined version group reported the highest score (mean 4.38 [SD 0.79]), which is significantly higher than the group receiving the empowerment intervention only (mean 3.91 [SD 0.7]). The second highest recommendation score is reported by those in the knowledge intervention only group (mean 4.27 [SD 0.87]).

### Perceived Impact of the App

Regarding participants’ perceived impact of the app on their knowledge, we found statistical differences among groups (*F*_2,137_=16.36; *P<*.001), with the combined interventions group reporting the highest impact (mean 4.70 [SD 0.72]), followed by the knowledge intervention group (mean 4.58 [SD 0.58]) and, finally, the empowerment intervention group (mean 3.89 [SD 0.88]). Regarding participants’ perceived impact of the app on their information-seeking behavior, the group receiving the knowledge and empowerment interventions combined reported the highest score (mean 4.34 [SD 0.91]), but we did not find any statistical differences between groups (*F*_2,137_=1.93; *P=*.15).

Regarding the participants’ perceived likelihood of an actual change in the vaccination behavior, only 1.4% of the participants reported that MorbiQuiz discourages vaccination, whereas 12.1% affirmed that it cannot make a difference (6 participants from the knowledge intervention group, 9 from the empowerment intervention group, and 2 from the combined interventions group). The large majority (86.5%) reported that the app could make parents opt for vaccination (41 from the knowledge intervention group, 35 from the empowerment intervention group, and 45 from the combined interventions group).

### Study 2

#### Participants’ Characteristics

In total, 115 respondents accepted to participate in the telephonic interview. Of these, one did not provide a telephone number. Of the 114 telephone numbers received, 39 participants did not suggest a date and time to be called. We called 75 participants, of which 15 never answered the call. The final sample (N=60) included 21 participants from the knowledge intervention group, 15 participants from the empowerment intervention group, and 24 participants from the combined knowledge and empowerment interventions group.

**Table 3:** Participants’ characteristics (study 2, N=60).

Characteristic		Experimental group		
		1: Quiz only (n=21)	2: Videos and messages only (n=15)	3 Quiz + Videos and messages (n=24)
**Gender, n (%)**				
	Women	19 (90)	14 (93,5)	23 (96)
	Men	2 (10)	1 (6,5 )	1 (4)
Age, mean (SD)		33.61 (3.99)	34.4 (5.22)	33.34 (5.61)
**Nationality, n (%)**				
	Italian	21 (100)	15 (100)	23 (96)
	Brazilian	N/A^a^	N/A	1 (4)
**Education, n (%)**				
	University	14 (67)	8 (53)	18 (75)
	Secondary School	6 (28)	7 (46)	5 (21)
	Apprentice	1 (5)	N/A	1 (4)
**No. of children, n (%)**				
	1	18 (85)	12 (80)	17 (66)
	2 or more	3 (15)	3 (20)	7 (34)

^a^N/A: not available.

Most participants were women (56/60, 93%), in their early 30s (mean age 33.78 years), Italian nationals (59/60, 99%), and with 1 child (47/60, 78%). See Table 3 for participants’ characteristics. The themes extracted were grouped around those related to participants’ experience with the quiz and those related to participants’ experience with the videos and messages.

#### General Feedback

When asked about their general opinion of the app, participants spontaneously attributed a number of qualities to MorbiQuiz that covered a range of aspects, from its look to its contents. In general, participants defined the app as useful, innovative, and engaging and described their experience as fun and pleasant. Most participants reported that MorbiQuiz was highly convenient, meaning that it is handy, quick, nondemanding, noninvasive, easily accessible, and functional. They found the duration of the quiz a perfect match between a regular and gradual activity. Other remarks concerned its contents, defined as neutral/unbiased, complete, trustworthy, and rich. They also found the app simple, intuitive, clear, well structured, and captivating. Finally, participants described MorbiQuiz as highly educational and a useful tool that can help parents or soon-to-be parents to make a vaccination decision and stimulate one’s information seeking. Participants’ experiences with the app were grouped around 4 main themes, 2 related to the knowledge intervention and 2 related to the empowerment themes.

#### Experiences With the Quiz

When asked how the app helped them make a vaccination decision, participants in the intervention targeting knowledge and that targeting knowledge and empowerment felt that, after using MorbiQuiz, their decision was reinforced, they were more confident, more knowledgeable on the vaccination, and had less fear of the side effects. The majority also complained that the app did not provide links to external resources after each quiz, which could have helped them enrich their knowledge further. To ensure that the app could be useful beyond the 10 days of quiz, about a quarter of the participants suggested to create a database containing all information provided by the quiz that is accessible and constantly updated with news. About half of the participants suggested creating a similar app to inform parents about other vaccinations such as meningococcal vaccination.

#### Learning From Failure

The large majority of the participants who received the knowledge intervention reported that a major quality of MorbiQuiz is that it offers a novel way of learning about vaccination compared with the most traditional educational tools. Participants described their learning process through the app as an active one, whose main steps comprised receiving a question, seeking adequate information to answer appropriately, providing an answer and learning from the textual outcome of each answer. One participant stated:

I would receive a question and, often convinced of my answer which eventually would turn to be wrong, I would go and seek information on why I got it wrong. And thus...In that sense, in my opinion, it helps increasing one’s knowledge.

11053, knowledge intervention

Most participants also stressed that MorbiQuiz invites to seek information actively and that it does so in a gamified way. They reported that this mechanism makes sure that either in case of a correct or a wrong answer, the participant has a chance to learn. In the first case, he or she will learn from the source consulted and from the textual content, whereas in the second case, he or she will learn to question the information sources consulted and judge their credibility next time, learning from the textual content:

It’s a call to play, it’s a call to act. It’s so interesting to me, when you open the first question, I mean, we have so many tools now to navigate online and find the right answer, don’t we? Indeed, it invites you...To understand, read, analyze, right? Then you give your answer. If it’s right, fine. You are happy that what you had seen was correct, and you deepen your knowledge with the answer that you receive. If it’s wrong, then you start questioning the source that you had looked up, don’t you? This challenge needs to be stressed. This means putting yourself on the line, going to seek information, and finally getting active yourself.

11051, both interventions

Through the mechanism that provided a textual explanation after any right or wrong answers, most participants found that MorbiQuiz was effective in eliminating their doubts on the vaccination and in providing novel information, as illustrated in the quotes below:

[I was] not knowledgeable on the topic, I didn’t know...and answering, at the end of each answer it would say if the answer was correct or wrong, and it would provide an explanation to the question and those were really very...very useful, because I had certain doubts and those have...they all have been practically removed.

11097, knowledge intervention

The modality with the quiz followed by the explanation is undoubtedly very useful, because either in case of correct answer or wrong answer it offers anyway extra information compared to what you already know.

11194, both interventions

Around half of the participants reported that the quiz also helped them improve their information-seeking skills:

The quiz really enlightened me on aspects that...that I did not know, therefore some questions that I got wrong, it has really put me in the condition to better inform myself on those things that I really did not know...In this sense it has made me more informed.

11076, knowledge intervention

Participants appreciated the timeliness of the feedback they received from the quiz, indicating that, when they provided the answer, assessing their answer was quick and straightforward:

I have learnt many things, and this is the most important thing because even by making a mistake, there were anyway very clear explanations which gave you points of view...things that I absolutely didn’t know. Then it was very immediate as a thing...I mean rather simple the flow from questions to answers.

11056, both interventions

#### A Challenge Against Oneself

When asked how they perceived the app’s leaderboard, the majority of the participants reported to have looked at it regularly during the quiz session. However, what emerges from participants’ reports is that the presence of the leaderboard does not correspond to a feeling of racing with others but rather competing with oneself, as illustrated in the quotes below:

I simply played a game and, in this game, I collected information by receiving answers...Personally, I also like to race as a person, to confront myself...and...I mean, it was not a game against others. It was a game against myself.

11231, knowledge intervention

It has motivated me, I mean I asked myself...Am I the only one who gets them wrong? [laughs] I was interested in looking at it in the end because I made mistakes and then I would go and look for information on that.

11053, knowledge intervention

The majority of the participants found that the leaderboard added fun to the experience of collecting information and pushed to search more information to answer the next questions in a better way:

I was a bit broken when I saw I was behind in the rank because I could not answer the questions...but it was fun, and the idea of the leaderboard was very stimulating.

11042, both interventions

It was fun because you would try to do your best possible. The leaderboard definitely acts as a...push. In a playful way, obviously.

11113, both interventions

Few participants reported to feel a sense of social support through the leaderboard, reporting a feeling of not being alone:

I think [the leaderboard] was...it was important that other parents have participated and have done the quiz...I felt...How to say...Not alone, that’s it.

11197, knowledge intervention

#### Experience With the Video/Messages

When asked how the app helped them make a vaccination decision, participants in the empowerment intervention(s) reported different general feedback. In particular, those exposed to both the quiz and the videos/messages felt that, after using MorbiQuiz, they had more confidence in their decision and knew more on the vaccination. Those participants in the empowerment intervention, on the contrary, were less convinced that the app had made an impact on their decision. In a similar fashion, when we elicited their feedback on the usefulness of the videos and messages, participants reported opposite views.

#### A Mother Like Me

Participants who received the videos and messages mainly reported comments on the videos, in particular the first one (the main and longest one). Around half of them found the video to be very close to their experience and pushing them to look for more information:

I found the video very clear, very close to me. The fact that the protagonist is a mother makes it even closer to the everyday life of us, mothers, rather than a more informative video, how to say, that would be colder, more detached.

11194, both interventions

Participants found a similarity between the actress’ experience and their struggle to make a sound MMR vaccination decision for their children, reporting that the video appeared to be authentic and trustworthy:

I felt it was really made by a...by a regular mother, not by someone...how to say...I mean by a mother like me! So I have to say, it was really nice...She would talk about the same problems that all mothers and fathers have when they have to choose.

11036, empowerment intervention

Few participants reported that they found a similarity between the decisional process described in the video and their decision-making process.

It felt like being...When I made the decision...like in this case, I mean I saw myself in this mother who gather information on the decision to vaccinate her child or not. I really liked that it was a real mother who talked. The character is trustworthy, it’s real, and authentic.

11051, both interventions

Some participants felt the video contained a direct message from a mother to another mother, whereas others felt like following the character’s story:

I interpreted it as a thought from a mother to a mother. I mean, a mother who tells you what she wanted to do with her child, and gives her advice as a mother to another mother.

11066, both interventions

It felt like following the story of this mother. It felt a bit like knowing her, like you were personally following her […].

11109, both interventions

#### Need for Direction

Around half of the participants declared that they found the video not useful, in the sense that it did not add anything to their knowledge nor stated the direction of the main character’s decision. As an alternative, they reported a preference for a video that would rather present information on the vaccination, possible side effects, and main benefits, as echoed by the quotes below:

The video does not provide information about the vaccination, it only tells about her that...It does not provide information per se, I did not find it particularly useful. I don’t know why, I would have preferred a video with information, and then you use that information to answer the questions of the quiz.

11238, both interventions

Maybe I was expecting that the mother would say in the end “this is what I chose,” maybe I was expecting this...I don’t know, it could be that we are used to see in the movies...to see a finale, but this mother was rather...rather cautious, she would say “I collected information before deciding.”

11003, both interventions

Some parents suggested maintaining the narrative format but replacing the mother with experts or different parents with contrasting opinions. In this sense, some clearly stated that they would not use a tool that is only made to invite them to seek information.

If the videos were present or not that would not have made any difference. Cause you could see this mother talking, telling her experience, but...But I think if there were more videos with, say, different opinions, from different mother, that would have maybe been more...more instructive, more of a general picture...

11225, both interventions

I think it necessarily has to give some kind of information, beyond suggesting parents to seek information, I mean I cannot imagine an app that I simply access to hear “seek information, you have to look for information, yes, go and do it”.

11027, empowerment intervention

A small number of participants stressed the passive component of the videos, compared with the active characterization of the quiz:

Honestly, I was not enthusiastic about the videos. They were kind of redundant. I found more answers and more stimuli in the quiz, maybe because when we are asked a question, it is up to us to answer and it sticks to our head for a longer time, as we think about it to find the correct answer...we think about it longer. But the videos, being a passive thing, did not make me enthusiastic.

11042, both interventions

## Discussion

### Principal Findings

The scope of this mixed-method study was to evaluate 2 interventions delivered through a mobile phone app aimed at increasing parents’ knowledge about the MMR vaccination and their empowerment in the MMR vaccination decision. Both interventions were previously tested in an RCT. In particular, we were interested in capturing participants’ opinion regarding a number of qualities of the app, such as usability and usefulness, and in acquiring information on their broader experience with the tool. A quantitative and qualitative study was conducted to reach these goals.

A first main finding springing from both studies is that overall participants perceived the app as highly usable and useful to make a vaccination decision. However, the results of the survey showed that the 2 groups receiving the quiz (alone or together with the videos/messages) liked the app significantly better compared with the group that only received the empowerment intervention through videos/messages. Furthermore, participants receiving only the quiz reported higher scores for most app’s qualities compared with those receiving the videos/messages in addition to the quiz. Educational interventions are the most commonly cited interventions in the literature [8], which might signal that they are also the most common interventions parents are exposed to and which they are acquainted with. This might explain why the educational version of the app received higher ratings. This is also the first immunization app in the Italian language with educational purposes and the first attempt to empower parents about their vaccination decision through a mobile device [20-23]. Participants might not be familiar with empowering interventions delivered through a video format and administered through a mobile phone app.

The results of the interviews also shed more light on between-group differences detected for the app’s qualities, highlighting different experiences in relation to the type of intervention participants were exposed to. Parents’ qualitative reports indicate that the knowledge intervention (employing the quiz and using elements of gamification) was perceived as an active learning experience, compared with the videos, which in turn were perceived as the passive exposure to a story. Furthermore, those in the knowledge group highlighted a number of positive aspects relative to learning, praising the gamified way by which they could not only acquire new information and question their previous knowledge but also improve their information seeking skills.

Parents receiving the empowerment intervention, on the other hand, lamented the lack of factual information that they would expect from a video, highlighting the emotional burden such a call for a self-determined decision might entail. The interview results also showed that mothers liked to identify themselves with the main character of the videos, as they share similar experiences and difficulties. However, beyond recognizing similarities with the protagonist, identification did not seem to be associated by parents with important aspects related to their decision making regarding their child’s MMR vaccination.

These results are in line with previous findings that interventions using gamification have the potential to increase engagement and intrinsic motivation [24-26]. In particular, our study confirms previous findings that participation in gamified interventions was associated with users’ engagement [27-30], enjoyment of activities [31-33], increased task performance [33-35], higher empowerment [27], learning [36-42], and more positive attitude [28,36,43]. Our participants’ reports that they felt more convinced of their vaccination decision after participating in the quiz are also corroborated by a previous study that found gamification to be effective in reinforcing a behavior [42].

The findings of our evaluation study provide more explanation to the results of the previous RCT [13], which found that only the group receiving the knowledge intervention significantly increased their intention to vaccinate against MMR and their confidence in making a vaccination decision. The results of the qualitative study can contribute to explain why we did not find a significant effect of the empowerment intervention on parents’ vaccination intention and confidence. Parents need a clear direction or, at least, a comparison between different points of views on vaccinations. Excessively pressuring them to find vaccination-related information and to talk to different people—without providing factual information at the same time—might generate frustration and emotional distress. Indeed, different reviews of the evidence on the effectiveness of interventions aimed at increasing vaccination coverage point out that multicomponent interventions that have educational purposes should consider that the educational component alone might not determine large increase in vaccination acceptance but could smooth the progress of implementation of other components [2,8,44].

Finally, parents indicated to be aware of the impact the app can have on their decision making, with the large majority reporting it could potentially lead parents to opt for the vaccination. Users’ awareness of the goal and the high potential of an app are crucial for making an app trustworthy and worth downloading or being recommended [45,46].

### Limitations

Although the studies showed to be successful in providing new insights into parents’ perceptions of a novel immunization app, a number of limitations should be noted. A first limitation is that both studies’ samples were mainly composed of provaccination or unsure parents. Acquiring the report of more vaccination-skeptical parents might have led to different results. A second limitation has to do with the incentives we offered to parents once the survey was completed. This might have played a role when parents reported their rating of the app, as they might have given higher scores to obtain the incentives we promised. Finally, social desirability biases may have occurred during the telephonic interviews. As the interviews were conducted by the team that developed the app, parents might have been led to report a positive experience to please the researchers.

### Conclusions

This evaluation study showed to be useful not only to assess the 2 interventions beyond the results of the previous RCT where they were tested but also to understand participants’ experience with the tool and contents they were exposed to and collect self-reported data on their perceived usability and usefulness of this instrument. The results can inform the design of future, similar interventions with educational or empowering purposes, suggesting that empowering efforts be always accompanied by the provision of factual information. Using a narrative format that allows identification can be appropriate, as it was reported to be associated with a feeling of social support that is called for by a recent taxonomy of communication interventions to improve routine childhood vaccination [10]. This, however, should not be employed alone but rather together with the presentation of more points of views and notions regarding, for instance, the risks and benefits of the vaccination.

## Appendix

Multimedia Appendix 1 Interview grid.

## Abbreviations

MARS: Mobile App Rating Scale
MMR: measles-mumps-rubella
RCTs: randomized controlled trials
