# Supplementary material for: Evaluation of a Mobile Phone–Based Intervention to Increase Parents’ Knowledge About the Measles-Mumps-Rubella Vaccination and Their Psychological Empowerment: Mixed-Method Approach
Source: JMIR Mhealth Uhealth. 2018 Mar 7;6(3):e59. doi: 10.2196/mhealth.8263 (PMC5863009; doi:10.2196/mhealth.8263)
Supplement: Multimedia Appendix 1 [file mhealth_v6i3e59_app1.pdf]

Appendix 1. Interview grid

General impressions	What do you think of MorbiQuiz? What was your experience with this app?<br>Is there anything about MorbiQuiz that you liked particularly?<br>Anything that you did not like? Anything that annoyed you?
Perceived effects	What has changed in you after using MorbiQuiz? What was the effect of MorbiQuiz on you, if any?<br>To what extent has MorbiQuiz helped you make an MMR vaccination decision for your child?<br>What effects can MorbiQuiz have on other parents?<br>Why should parents download and use MorbiQuiz?
Quiz and gamification	To what extent has the quiz helped you improve your knowledge about the MMR vaccination?<br>What do you need to feel more knowledgeable?<br>How did you perceive the leaderboard?
Videos, messages and interpersonal communication	What feelings did you have after watching the video? Which thoughts came to your mind after watching the video? How did you feel about receiving Sofia's messages?<br>In your opinion, what is the take-home message of the video?<br>To what extent videos and messages helped you feel more empowered in your decision?<br>What do you need to feel more empowered?
Suggestions	How would you improve MorbiQuiz?<br>Which features would you add/remove?<br>How would you see MorbiQuiz in the future?
